# Structural Characterization of La_0.6_Sr_0.4_CoO_3−*δ*_ Thin Films Grown on (100)-, (110)-, and (111)-Oriented La_0.95_Sr_0.05_Ga_0.95_Mg_0.05_O_3−*δ*_

**DOI:** 10.3390/ma17081802

**Published:** 2024-04-14

**Authors:** Sergej Ražnjević, Sandra Drev, Andreas E. Bumberger, Maxim N. Popov, Matthäus Siebenhofer, Christin Böhme, Zhuo Chen, Yong Huang, Christoph Riedl, Jürgen Fleig, Miran Čeh, Markus Kubicek, Zaoli Zhang

**Affiliations:** 1 Erich Schmid Institute of Materials Science, Austrian Academy of Sciences, Jahnstraße 12, 8700 Leoben, Austria; zhuo.chen@oeaw.ac.at (Z.C.); yong.huang@oeaw.ac.at (Y.H.);; 2 Center for Electron Microscopy and Microanalysis, Jožef Stefan Institute, Jamova cesta 39, 1000 Ljubljana, Sloveniamiran.ceh@ijs.si (M.Č.); 3 Institute of Chemical Technologies and Analytics, TU Wien, Getreidemarkt 9, 1060 Vienna, Austria; andreas.bumberger@tuwien.ac.at (A.E.B.); christin.boehme@tuwien.ac.at (C.B.); christoph.riedl@tuwien.ac.at (C.R.); markus.kubicek@tuwien.ac.at (M.K.); 4 Materials Center Leoben Forschung GmbH (MCL), Roseggerstraße 12, 8700 Leoben, Austria; maxim.popov@mcl.at; 5 Centre of Electrochemistry and Surface Technology, Viktor Kaplan-Straße 2, 2700 Wiener Neustadt, Austria; 6 Department of Nuclear Science and Engineering, Massachusetts Institute of Technology, 77 Massachusetts Avenue, Cambridge, MA 02139, USA

**Keywords:** perovskite, brownmillerite, thin films, electron energy loss spectroscopy, transmission electron microscopy, strain, ordering phenomenon

## Abstract

In this study, a detailed structural characterization of epitaxial ${\text{La}}_{0.6}$${\text{Sr}}_{0.4}$${\text{CoO}}_{3-\delta }$ (LSC) films grown in (100), (110), and (111) orientations was conducted. LSC is a model air electrode material in solid oxide fuel and electrolysis cells and understanding the correlation of bulk structure and catalytic activity is essential for the design of future electrode materials. Thin films were grown on single crystals of the perovskite material ${\text{La}}_{0.95}$${\text{Sr}}_{0.05}$${\text{Ga}}_{0.95}$${\text{Mg}}_{0.05}$${\text{O}}_{3-\delta }$ cut in three different directions. This enabled an examination of structural details at the atomic scale for a realistic material combination in solid oxide cells. The investigation involved the application of atomic force microscopy, X-ray diffraction, and high-resolution transmission electron microscopy to explore the distinct properties of these thin films. Interestingly, ordering phenomena in both cationic as well as anionic sublattices were found, despite the fact that the thin films were never at higher temperatures than 600 °C. Cationic ordering was found in spherical precipitates, whereas the ordering of oxygen vacancies led to the partial transition to brownmillerite in all three orientations. Our results indicate a very high oxygen vacancy concentration in all three thin films. Lattice strains in-plane and out-of-plane was measured, and its implications for the structural modifications are discussed.

## 1. Introduction

Mixed ionic electronic conducting (MIEC) oxides are an important material class for solid oxide electrolysis or fuel cells (SOEC/SOFC), for catalysis or sensors [1,2,3]. There has been a notable focus on the advancement of MIEC materials for SOFC cathodes, with a focus on perovskites, double perovskites, Ruddlesden–Popper phases, and other layered oxide materials [3]. Among those, cobaltite-based perovskite thin films (such as LSC and ${\text{La}}_{1-x}$${\text{Sr}}_{x}$${\text{Co}}_{1-y}$${\text{Fe}}_{y}$${\text{O}}_{3-\delta }$) [3] are of particular importance due to their high electronic as well as ionic conductivity [3,4,5,6]. By that and their high catalytic activity as oxygen electrodes, they enable a considerable reduction in operation temperature which is a major aim for solid oxide cells [7]. In addition, as thin films, they are also a very well investigated model system towards defect chemistry [8], modifications or degradation of the surface [9], or structural transitions [10], effects that ultimately may even be interconnected. For example, growing (La,Sr)${\text{CoO}}_{3}$ thin films under tensile strain may lead to considerably enhanced oxygen vacancy concentration that may further lead from perovskite (${\text{ABO}}_{3}$) to brownmillerite (BM, ${\text{ABO}}_{2.5}$) phase transitions [10,11]. A detailed study of the structure-property relations is hence important for enhancing our understanding towards further improving the catalytic properties of LSC. However, high-quality epitaxial growth directly on electrolyte materials is not easily possible as typical oxide electrolytes such as Y:${\text{ZrO}}_{2}$ and Gd:${\text{CeO}}_{2}$ have a fluorite structure.

In this study, we turn to strontium- and magnesium-doped lanthanum gallate (LSGM) [12,13,14] single crystals cut in three different orientations (100), (110), and (111) as a substrate. LSGM is a perovskite that allows the epitaxial growth of LSC with moderate tensile in-plane strain. Furthermore, LSGM is an excellent oxygen ion electrolyte, and thus the system LSGM|LSC also enables a direct electrochemical characterization, which was performed in a separate study [15]. In that study, we could show that the oxygen exchange kinetics on pristine LSC surfaces did not strongly depend on crystal orientation. However, their degradation behavior was distinctly different, with (100)-oriented LSC undergoing a significantly faster degradation due to sulfate adsorption, compared to the other two orientations. LSGM exhibits a high ionic conductivity of oxygen ions [12,16,17], and its electronic properties vary with the A- and B-site doping levels [18]. Based on the doping level, the crystal symmetry may also change from the ideal perovskite lattice. For example, investigations at room temperature of the ${\text{La}}_{1-x}$${\text{Sr}}_{x}$${\text{Ga}}_{1-2x}$${\text{Mg}}_{2x}$$0_{3-y}$ system (x = 0.05, LSGM-05, and x = 0.1, LSGM-10) showed that the LSGM-05 had an orthorhombic symmetry, while LSGM-10 had a monoclinic symmetry [13,19].

Over the past few decades, pulsed laser deposition (PLD) has emerged as a highly effective method for producing high-quality thin films while accurately reproducing the target stoichiometry [20,21,22]. PLD deposition involves numerous parameters that significantly influence the characteristics of the resulting film, including laser fluence, background gas pressure, and target-to-substrate distance [23]. It has been found that, for example, high laser fluence can lead to stoichiometry deviations [24]; however, by precise control of the parameters, stoichiometric growth is possible and usually more easily achieved than with sputtering methods [25].

In this work, we investigated the structural properties of ${\text{La}}_{0.6}$${\text{Sr}}_{0.4}$${\text{CoO}}_{3-\delta }$ on (100)-, (110)-, and (111)-oriented ${\text{La}}_{0.95}$${\text{Sr}}_{0.05}$${\text{Ga}}_{0.95}$${\text{Mg}}_{0.05}$${\text{O}}_{3-\delta }$ (LSGM) substrates. Lattice parameters as well as the symmetry of the LSGM substrate varied between the differently oriented samples. Due to epitaxial growth, strain dynamics within the thin film were examined using high-resolution HAADF images and geometrical phase analysis. Our observations indicated a selective in-plane strain relaxation, and a pronounced out-of-plane relaxation for all three orientations. By investigating all three orientations, we gained deeper insight into both the elastic properties as well as the consequences in defect chemistry such as the tendency towards ordering phenomena.

## 2. Experimental Procedure

Pulsed laser deposition was used to deposit 40, 80, and 120 nm of LSC on (100)-, (110)-, and (111)-oriented LSGM substrates. The deposition was carried out at 600 °C, 0.04 mbar ${\text{O}}_{2}$ atmosphere, a laser frequency of 2 Hz, and a fluence of 1.1 J/${\text{cm}}^{2}$. In all three samples, the thin film had a so-called cubic-to-cubic orientation with respect to the substrate (i.e., (010${)}_{\mathrm{LSC}}$||(010${)}_{\mathrm{LSGM}}$ and [010${]}_{\mathrm{LSC}}$||[010${]}_{\mathrm{LSGM}}$). Methods describing substrate preparation can be found elsewhere [26].

Samples were thinned for the transmission electron microscope (TEM) analysis using two different techniques. First, 80 nm LSC/(110) LSGM and 80 nm LSC/(111) LSGM samples were prepared by the standard FIB-cut technique on a Thermo Fisher Scios 2 DualBeam FIB/SEM, which employs a Ga-ion beam at 30 kV accelerating voltage. Second, both 40 and 80 nm LSC/(100) LSGM were prepared using a conventional sample preparation technique where the samples were sawed to slices of 2 mm in thickness and glued into a “sandwich” creating the cross-section. The samples were thinned by grinding to a thickness of 100 µm. Then, the samples were further thinned down to 10 µm using a dimple grinder. Finally, the sample was thinned to an electron transparent thickness using an Ar-ion milling system (Gatan—Precision ion polishing system).

The surface morphology was investigated using a Bruker Nanoscope V multimode in the AFM tapping mode on an area of 1 µm × 1 µm. Three-dimensional AFM images were obtained using the Gwyddion software (Version 2.64) [27]. X-ray diffractometry (XRD) analysis was performed on a Malvern-Panalytical Empyrean X-ray diffractometer equipped with a GaliPIX3D detector and a hybrid monochromator. The θ–2θ scan was performed on 120 nm samples in all three orientations.

Transmission electron microscopy (TEM) techniques were performed using a probe-corrected JEOL ARM 200F operating at 200 kV to analyse samples with 80 nm of LSC deposited. Micrographs were recorded using a high-angle annular dark-field (HAADF) detector with a 68–280 mrad collection semi-angle range. An image-side Cs-corrected JEOL 2100F, also operating at 200 kV, was used to analyse the 40 nm LSC/(100) LSGM sample. Here, a Gatan Orius camera SC1000 was used to record micrographs. Strain states of the samples were determined from the HAADF micrographs by employing the geometrical phase analysis (GPA) procedure in the Gatan DigitalMicrograph software (Version 3.50.3584.0) [28]. In order to determine the in-plane and out-of-plane lattice parameters of the samples, a standard oriented gold sample provided by Agar Scientific was used to calibrate the micrographs.

Electron energy loss spectroscopy (EELS) measurements were performed on a Jeol 2100F microscope equipped with a tridiem system, and on a Jeol ARM 200F equipped with a dual EELS quantum spectrum-imaging filter. Line-profile EELS measurements were performed in the STEM mode using the Jeol 2100F microscope. Collection and convergence semi-angles were set to 125 mrad and 7.5 mrad, respectively, and a dispersion of 0.5 eV per channel was used. The energy resolution was set to 2.1 eV. The signal was filtered using the principal component analysis (PCA), which is a built-in feature in the Gatan Microscopy Suite. The white-line ratio was estimated using the Pearson model [29,30,31,32]. In this model, the area under $L_{2}$ and $L_{3}$ is integrated and a step function is used to remove the background. The height ratio of the steps is deliberately set at 2:1 to accurately mirror the 2p spin-orbit split states.

The enthalpy of formation for monoclinic and tetragonal LSC/(100) LSGM was determined by ab initio calculations using the VASP code [33,34,35,36], with the PBEsol [37] exchange-correlation functional. Basis set expansion was truncated using the energy cutoff of 520 eV, while the Brillouin zone integration was performed on a uniform Γ-centred mesh with 2 × 8 × 8 k-points. The employed lattice parameters were derived utilizing lattice parameters derived from TEM imaging. Specifically, for the tetragonal phase, the in-plane lattice parameter was set to 0.393 nm and the out-of-plane lattice parameter was set to 0.389 nm. In contrast, for the monoclinic phase, we set the in-plane and out-of-plane lattice parameter to 0.401 nm and 0.390 nm, respectively, with the angle β set to 89.3°. For each phase, a supercell was constructed by replicating the unit cell five times along the a-axis, and within that supercell, three La atoms and two Sr atoms were distributed in two ways (see Tables S1–S4 in the Supplementary Materials for more detail). Ionic relaxations to ensure residual forces not exceeding 0.01 (eV/Å) were permitted within the supercell while preserving the cell lattice parameters and angles. Additionally, a ferromagnetic spin ordering was applied to the Co atoms.

## 3. Results And Discussion

**Figure 1:**
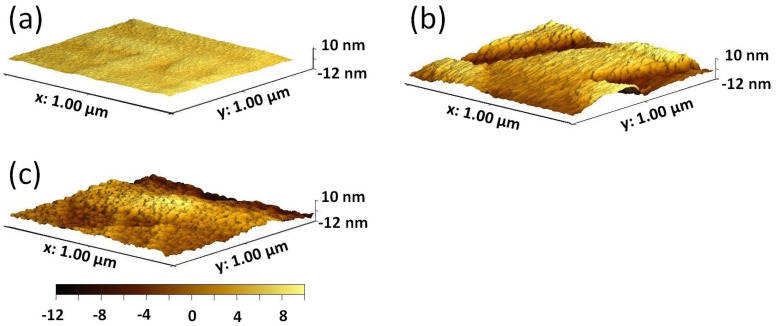
Atomic force microscopy images of the surface morphology of a 120 nm thick layer of LSC/(100) LSGM (**a**), LSC/(110) LSGM (**b**), and LSC/(111) LSGM (**c**).

The surface morphology of the three samples is illustrated in Figure 1, where distinct characteristics are observed. The RMS roughness of the samples is similar and quite low, with the values of 0.5 nm, 0.5 nm, and 1.0 nm for the (100)-, (110)-, and (111)-oriented samples, respectively.

**Figure 2:**
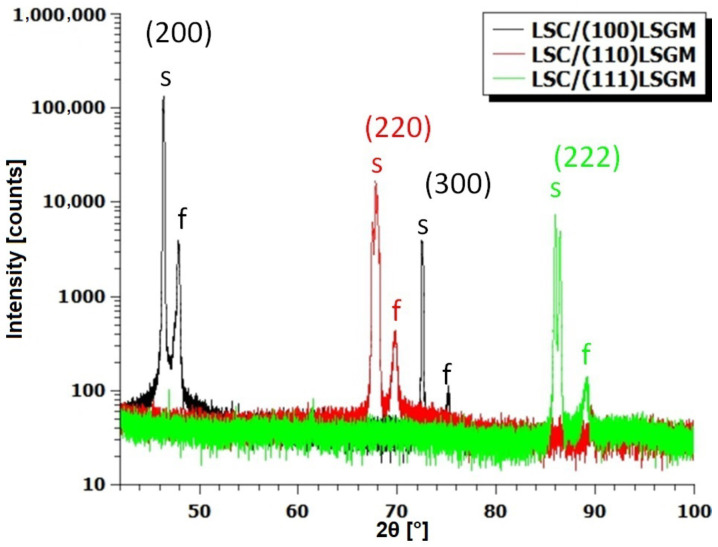
XRD θ–2θ scan of a 120 nm thick layer of the LSC/(100) LSGM, LSC/(110) LSGM, and LSC/(111) LSGM. Substrate peaks are marked with the letter s, while the LSC peaks are marked with the letter f.

Figure 2 shows a θ–2θ scan of the three samples. The diffractogram is composed of the contributions of the LSGM substrate (s), and the LSC thin film (f) reflections. The reflections from an LSGM substrate are not sharp; instead, they appear as a composite of multiple contributions, suggesting the existence of domains within the substrate. In line with Bragg law, these domains vary in their d-spacing. LSGM peaks in LSC/(100) LSGM and LSC/(110) LSGM samples are composed of contributions of seven different domains, while the LSGM peak in the LSC/(111) LSGM comprises of only two domains. The close-up of these domain contributions to the peak intensity can be seen in Figure S1 in the Supplementary Materials section.

Table 1 shows the lattice spacings of the LSGM domains obtained by the θ-2θ scan.

The conventional approach of TEM-micrograph calibration uses the d-spacing measurements of the substrate. However, because the TEM lamellae represent only a small fraction of the entire sample scanned in XRD, and considering the presence of domains with varying d-spacing in LSGM, we used the standard oriented gold sample for the TEM-micrograph calibration. Table 2 shows the LSGM lattice spacings of the three samples, as well as their crystal system, obtained from the TEM micrographs. (100) LSGM had a monoclinic crystal system (β = 89.3°), while (110) LSGM and (111) LSGM had a tetragonal (pseudo-cubic) crystal system. These values did not match the expected values of the lattice spacings of the LSGM substrate from the literature [26], but we want to stress that, while reported XRD measurements were averaged over different crystal domains, TEM only probed a very limited region of the sample. Further investigations of the cause of the LSGM crystal instabilities are beyond the scope of this paper.

Fully relaxed LSC has a lattice parameter of $a_{\mathrm{pc}}=\left(3.838\pm 0.003\right)$ Å [38]. Therefore, we can estimate what the strain of the LSC thin films would be if we assume a fully pseudomorphic growth by comparing this value with the values obtained in Table 2. The strain state estimation can be found in Table 3.

**Figure 3:**
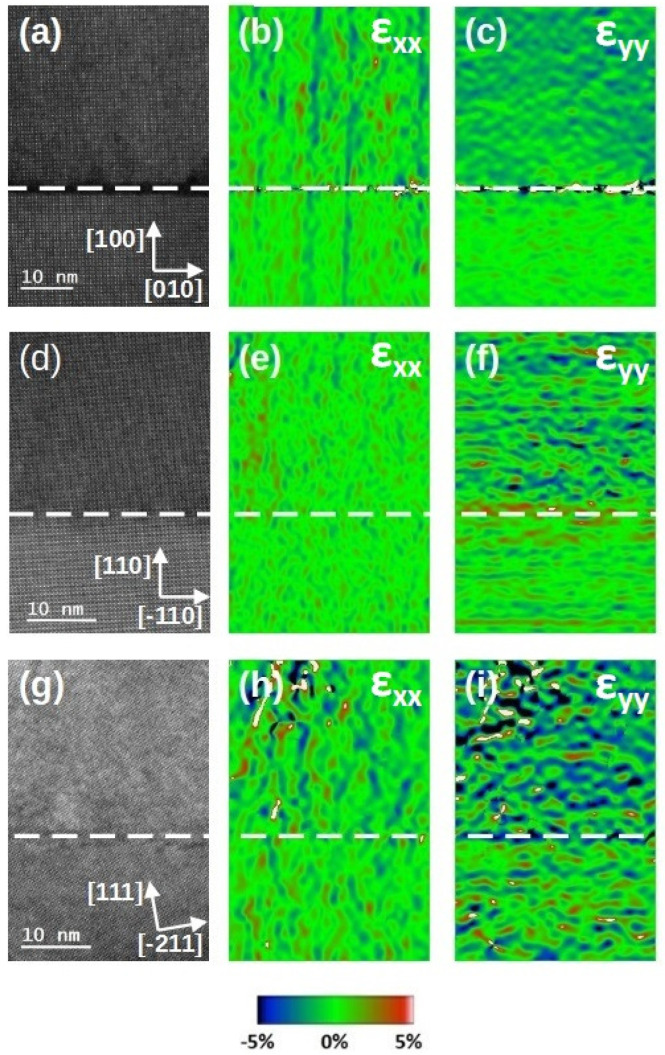
(**a**,**d**,**g**) shows HAADF images, (**b**,**e**,**h**) shows the in-plane $\epsilon_{xx}$ strain map, (**c**,**f**,**i**) shows the out-of-plane $\epsilon_{yy}$ strain map of LSC/(100) LSGM, LSC/(110) LSGM and LSC/(111) LSGM, respectively, with a 80 nm thick LSC layer. Interfaces are marked with a white dashed line.

Figure 3 shows medium-magnified Z-contrast STEM images of the three samples with their corresponding in-plane and out-of-plane strain maps. HAADF images confirmed that all three samples had an epitaxial growth. However, in the LSC/(100) LSGM sample, a dark contrast area was present all along the interface. This dark contrast at the interface corresponded to an amorphous phase of the LSC thin film. The amorphous phase was most likely introduced during the Ar-ion bombardment during the sample preparation procedure. Even though the image contrast of this phase strongly resembled the contrast observed in the premelting phenomena at the Rudelsden Popper (RP) faults of the perovskite-type ${\mathrm{BaCeO}}_{3}$ in the work by Kim et al. [39], here, it was not reasonable to assume the premelting phenomena to be the driving force of the amorphous phase formation due to the fact that the highest temperature this sample experienced was not more than 600 °C. To further investigate these experimental observations, we conducted ab initio calculations. Assuming that LSC fully adapted to the lattice parameters of LSGM, we estimated these oxides’ formation enthalpy (FE) and found that the FE of the tetragonal LSC phase was 0.2–0.3 eV/f.u. lower than that of the monoclinic LSC phase. Note that the monoclinic phase exhibited parameters that imposed more strain compared to the bulk material than the tetragonal phase did. The lower formation enthalpy indicated a more stable atomic arrangement, making the structure more resilient against the disruptive effects of Ar-ion bombardment. This provided a theoretical explanation of why the amorphisation under identical conditions occurred in monoclinic LSC rather than in the tetragonal LSC phase.

The orientation of the terminating plane of the substrate was nicely defined for [100] and [110] orientation. However, for the [111] orientation, there was a miscut of about 10.3° with respect to the surface normal.

**Figure 4:**
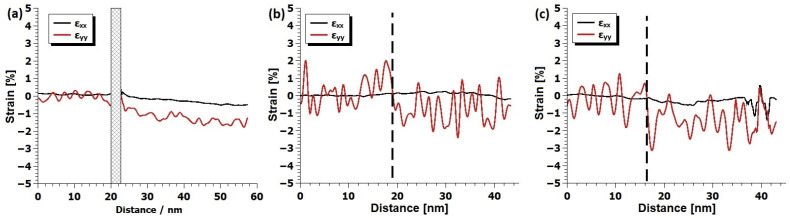
Area profiles of strain profiles from Figure 3. For the (100)-oriented sample (**a**), the interface region is hidden since the GPA analysis was not possible due to the lack of contrast. For (110) (**b**) and (111) (**c**), the interface position is highlighted with dashed black lines. In all three cases, the direction of area profiles is from the substrate towards the film.

In Figure 4, area profiles of the strain maps from Figure 3 are illustrated. In this context, the mean strain values of the substrate were intentionally set to zero, facilitating the measurement of the thin film’s strain state in relation to the substrate. In the LSC/(100) LSGM sample, both in-plane and out-of-plane strain relaxation were evident throughout the layer thickness. Similarly, the LSC/(111) LSGM exhibited strain relaxation in both directions, though the strain profile appeared more irregular. Conversely, for the LSC/(110) LSGM, strain relaxation was observed solely in the out-of-plane direction, with no significant relaxation in the in-plane direction.

**Figure 5:**
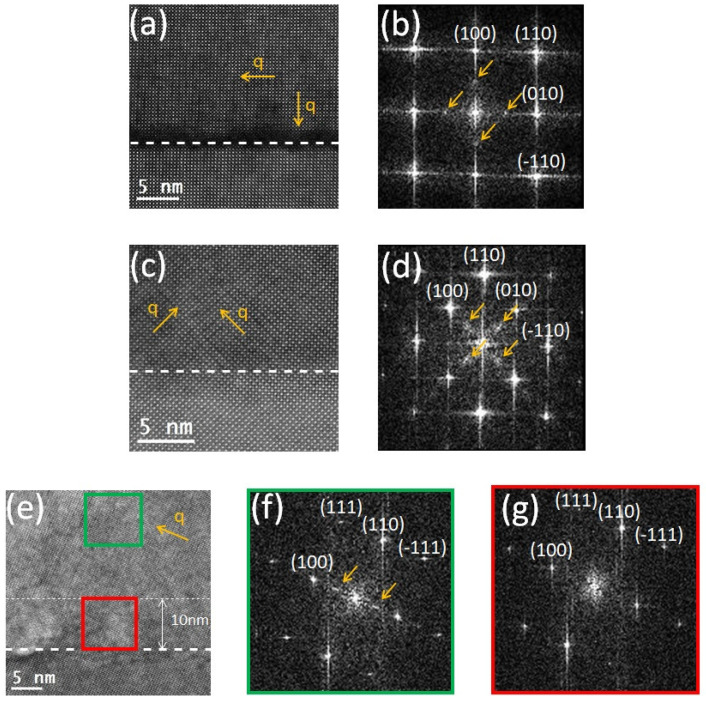
(**a**,**c**,**e**) show HAADF images of 80 nm thick LSC on (100) LSGM, (110) LSGM and (111) LSGM, respectively. (**b**,**d**) are FFT patterns of the film area in images (**a**) and (**c**), respectively. Images (**f**,**g**) are FFT patterns of different areas of the film in the image (**e**); the FFT pattern shown in the image (**f**) corresponds to the region (green rectangle) further away from the interface, while image (**g**) shows the FFT pattern in close proximity to the interface (red rectangle) where the oxygen-ordering free zone exists. White dashed lines mark the interfaces. In the image (**e**), the end of the oxygen-ordering free region is marked by a thin, white dashed line 10 nm away from the interface. Yellow arrows mark the direction of the modulation vector q.

Previous works have shown that under strain, LSC thin films may undergo oxygen-vacancy ordering and form a brownmillerite structure [40,41,42]. Figure 5 shows the atomic-resolution HAADF images of the three samples together with their corresponding FFT patterns. Both LSC/(100) LSGM and LSC/(110) LSGM have a brownmillerite ordering in both the [100] and [010] directions. However, in the LSC/(111) LSGM sample, ordering is visible only in the [100] direction. Here, it is not clear whether or not the ordering is present in the [010] direction since the [0–11] view direction only allowed us to examine the [100] direction. The similarity of all thin films in terms of oxygen content and ordering (see also the EELS analysis below) in the upper film regions is in agreement with our electrochemical results [15], which found no significant differences between the (oxygen vacancy-mediated) oxygen exchange kinetics of pristine, differently oriented films. Furthermore, in the LSC/(111) LSGM sample, there was a 10 nm thick region in which there was no brownmillerite ordering present. That region spanned all along the interface of the sample and in this paper, we refer to it as a “free region”, while for the region above with a BM ordering, we refer to it as the “film region”. FFT patterns were examined and they further confirmed the presence of the brownmillerite ordering by the half-integer reflections in the [100] or [010] directions. It is clear from Figure 5b,d that the ordering is present in both directions simultaneously. In Figure 5f,g FFT patterns taken from the film region (green) and from the free region (red) are shown. The FFT-pattern taken from the free region lacks half-integer reflections, which further confirms that there is no oxygen-vacancy ordering present.

**Figure 6:**
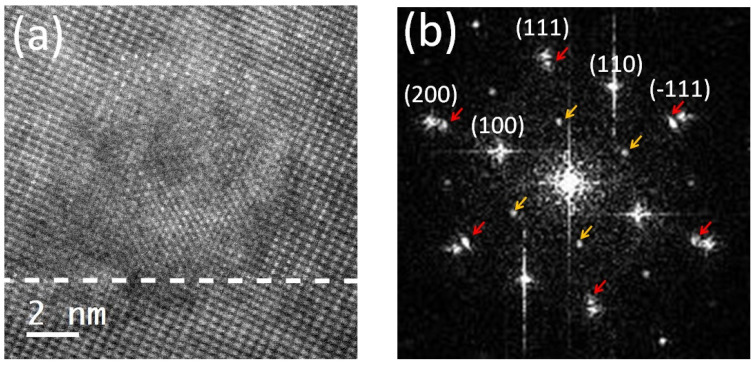
(**a**) shows an HAADF image of the precipitate just above the interface (highlighted with a white dashed line) of the 80 nm LSC on the (111) LSGM sample. (**b**) shows the FFT pattern of the entire (**a**) image. Yellow arrows mark half-integer reflections $\left(\frac{1}{2}\frac{1}{2}\frac{1}{2}\right)$ while the red arrows denote splitting of the (111) and (200) reflections.

Figure 6 shows the presence of precipitates in the free region of the LSC/(111) LSGM sample. The shape of the precipitate is round with a diameter of around 10 nm so it spans across the whole free-region thickness. The precipitate has the same structure and orientation as the film and the substrate. However, a lattice parameter change can be observed in the FFT pattern (Figure 6b), where the split reflections are highlighted by red arrows. The difference in the lattice parameters of the film and the precipitate is quite significant, where the precipitate has an 8.81% increase compared to the thin film in the free region. Additionally, a doubling of the lattice parameters in the [111] direction can be seen (highlighted by yellow arrows). The population of these precipitates is about one per 70 nm, but their distribution is not uniform, as can be seen from Figure S4 in Supplementary Materials, where the precipitates were recorded in a low-magnification mode using the dark-field imaging technique. Due to the strong lamella bending, precipitates are only visible on the right side of the image and are highlighted by red arrows. From the HAADF micrograph (Figure 6), it can be seen that the intensity at the A-site locations has an ordered structure, which is responsible for a [111] doubling. An intensity line trace over the atom column, given in Figure S2 in Supplementary Materials, further confirms the [111] ordering. We assume that the doping level of Sr is being modulated in that precipitate, so that the columns with more Sr scatter less intensively than the columns with less Sr (more La), which explains the intensity ordering in the mass-sensitive (Z contrast) HAADF image. A similar analysis was conducted on a Pr,${\mathrm{BaCoO}}_{3-\delta }$ in the work by Jin et al. [43] where they found the intensity modulation in the Co-O planes.

In order to examine the chemical properties of the three films, an electron energy loss analysis was performed. Due to the fact that the measurements were performed on different systems and in different conditions, a quantitative (i.e., white-line ratio) comparison between samples was not an option. However, a relative peak shift analysis was performed. It has been shown that the edge onset difference may be correlated to the oxidation state of some systems (with an increasing energy onset difference, the oxidation state of the system becomes higher) [44,45]. The onset of the oxygen K-edge and Co L-edge was determined using the method proposed by Tan et al. [44], in which the onset is determined as a point with 10% of the edge maximum intensity to prevent the influence of a possible prepeak effect and noise on the onset position. From Figure S6 in the Supplementary Materials, it was determined that the energy onset difference ΔE of the (100)- and (111)-oriented sample was the same, with a value of (247.7 ± 0.5) eV, while the energy onset difference of the (110)-oriented sample was a bit larger with a value of (248.5 ± 0.5) eV. Taking into the account the error of 0.5 eV, this slight increase is negligible. Thus, we conclude that the oxidation state of all three films is the same, just as expected from the brownmillerite oxidation $O_{2.5}$. Furthermore, measurements revealed that the peaks in the oxygen K-edge exhibited a noticeable shift relative to each other across all three orientations. This is not surprising since the oxygen K-edge is mainly dependent on the crystal geometry, and in these systems, the strain state is different [44].

**Figure 7:**
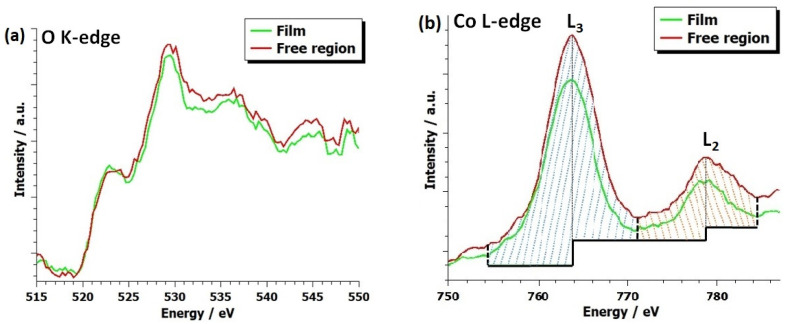
STEM-EELS spectra of 80 nm LSC on (111) LSGM showing the oxygen K-edge (**a**) and Cobalt $L_{2,3}$-edge (**b**). In (**b**), the baseline is marked by a thick black step function, thick black dashed lines showcase the integration range of the $L_{2}$ and $L_{3}$ edges, and thin black dashed lines denote the edge maximum, which are also the positions of the steps in the step function. Dashed blue and dashed orange lines showcase the integration area for the $L_{3}$ and $L_{2}$ edges in the free region, respectively.

It has been shown that due to the electron beam irradiation, oxygen vacancies may assert random positions in the LSC film and this random vacancy distribution is stable [10]. Therefore, using a STEM line-scan, we probed whether the first 10 nm of the LSC/(111) LSGM thin film had higher oxygenated ${\mathrm{CoO}}_{x}$ planes, or whether oxygen vacancies were being randomly distributed while keeping the brownmillerite oxidation level. Three peaks are present in the oxygen K-edge in Figure 7, both for the free region and the film region. Overall, the total intensity of the K-edge of the free region was higher than the intensity taken from the film region. A similar signal increase was also present in the Co L-edge, and we attributed this signal increase to the thickness variation of the sample. However, the thickness variation was not more than 0.2 (t/λ) between the free region and the film, so the multiple scattering effect was not removed using a Fourier ratio deconvolution. Furthermore, it has been shown that the oxygen content in the Co planes is closely related to the Co white-line ratio. Estimated values of the Co $\frac{L_{3}}{L_{2}}$ white-line ratio were 2.53 ± 0.13 and 3.24 ± 0.13 for the free region and the film, respectively. In previous works, it has been shown that with the increasing white-line ratio, the Co valence state decreases [31,46,47]. This indicates that the oxygen content in the LSC/(111) LSGM is not uniformly distributed, but that it has a higher concentration in the first 10 nm where there is no oxygen-vacancy ordering present.

**Figure 8:**
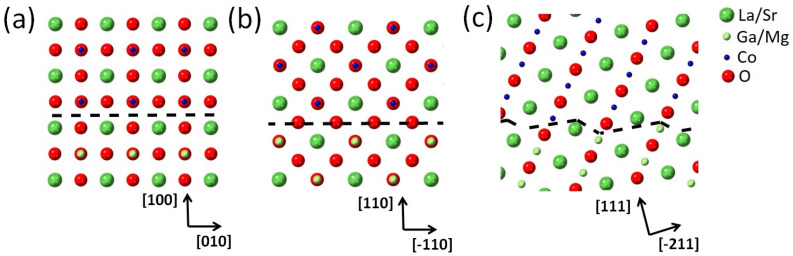
(**a**–**c**) show the models of the atomic structures at the interface of 40 nm LSC on (100) LSGM, and 80 nm LSC on (110) LSGM and (111) LSGM, respectively. The interface is highlighted by a black dashed line. (**a**,**b**) are shown in the [001] zone axis while (**c**) is shown in the [0–11] zone axis.

To emphasize the interface structure, atomic models are proposed in Figure 8. In the LSC/(100) LSGM sample, the termination plane of the substrate was an A-plane (La/Sr-plane). This was analysed from an HRTEM image of 40 nm LSC/(100) LSGM, presented in Figure S3 in the Supplementary Materials due to the presence of the dark contrast in the HAADF micrographs of the 80 nm thick sample. In the LSC/(110) LSGM sample, there was an AB-plane and a pure oxygen plane; hence, in between the AB-plane of the LSGM substrate and the LSC film, there was an oxygen plane. If the oxygen plane was not in between the two AB planes, that would be visible in the sudden d-spacing increase due to the Coulomb interaction (similar to the d-spacing increase over the oxygen-deficient Co plane [10]).

The interface position was determined by examining the intensity line profile (averaged over 23 atomic distances) where the interface position was set halfway in between the two regions with a different background intensity (this background intensity difference arises from the fact that LSGM, which has more heavy La atoms, is more resilient to Ar ion bombardment, so the LSGM region is slightly thicker than the LSC region, hence the difference in the background). Figure S5 in the Supplementary Materials shows the region in the HAADF image as well as the intensity line profile used for the interface analysis. Finally, for LSC/(111) LSGM, there is no single terminating plane because of the substrate miscut. As shown in Figure 8c, the substrate and the film have a step-like interface where, in one step, the substrate is terminated by a B-plane and, in another, by an A-plane, and this step-like interface is repeated over and over through the sample.

## 4. Conclusions

In this study, we investigated the growth of the LSC thin films on (100)-, (110)- and (111)-oriented LSGM substrates. We showed that LSC films grew epitaxially on all substrates; however, distinct differences and ordering phenomena existed. We ascribed those differences to the introduction of in-plane tensile strain on the one hand and to a domain-like structure of the substrates found with both XRD and TEM on the other hand. In LSC/(100) LSGM, the substrate had a monoclinic symmetry with a tilt angle of 89.3°, whereas in LSC/(110) LSGM and LSC/(111) LSGM, the substrate had a tetragonal symmetry. Oxygen-vacancy ordering towards a brownmillerite formation was observed in all three orientations, and where detectable, it occurred in both [100] and [010] directions. This similarity in terms of oxygen content and ordering also agreed with previous electrochemical studies, which found similar oxygen exchange kinetics on pristine, differently oriented LSC thin films. In LSC/(111) LSGM, in contrast to the other orientations, there was a region about 10 nm thick all along the interface, which exhibited no oxygen-vacancy ordering. The EELS analysis showed that in that free region, there was a higher oxygen concentration than in the rest of the film. Additionally, in that region, several spherical precipitates of about 10 nm diameter were present that had an A-site cationic ordering together with significantly larger lattice parameters than the surrounding thin film. These unique structures occurring only in the (111) orientation may help in strain accommodation.

## Figures and Tables

**Table 1:** d-spacings of the (100), (110), and (111) LSGM single crystals determined from the different domain contributions in the XRD θ–2θ scans of samples with 120 nm thick LSC layers.

Domain	$d_{100}$ / Å	$d_{110}$ / Å	$d_{111}$ / Å
A	3.919	2.775	2.260
B	3.915	2.772	2.250
C	3.913	2.763	
D	3.911	2.761	
E	3.908	2.756	
F	3.906	2.754	
G	3.900	2.748	

**Table 2:** d-spacings and crystal systems for the (100), (110) and (111) LSGM single crystals determined from TEM micrographs of samples with a 80 nm thick LSC layer.

Direction	(100) LSGM	(110) LSGM	(111) LSGM
d-spacing / Å	$d_{010}$ = 4.01	$d_{\bar{1}10}$ = 2.78	$d_{\bar{2}11}$ = 1.60
	$d_{100}$ = 3.90	$d_{110}$ = 2.75	$d_{111}$ = 2.23
Crystal system	Monoclinic	Tetragonal	Tetragonal

**Table 3:** Estimated in-plane $\epsilon_{xx}^{est}$ and out-of-plane $\epsilon_{yy}^{est}$ lattice parameter deviation of LSGM with respect to bulk LSC as determined from samples with a 80 nm thick LSC layer.

Orientation	$\epsilon_{\mathrm{xx}}^{\mathrm{est}}$ / %	$\epsilon_{yy}^{\mathrm{est}}$ / %
(100)	4.48	1.62
(110)	2.45	1.33
(111)	2.11	0.63

## Data Availability

Data are contained within the article and Supplementary Materials.

## Supplementary Materials

The following supporting information can be downloaded at: https://www.mdpi.com/article/10.3390/ma17081802/s1, Figure S1: Close up view at the (a) (200), (b) (220) and (c) (222) reflections from the XRD Θ -2Θ scan; Figure S2: Average background subtraction filtered HAADF image (left) and the intensity line trace (right). In the line-trace red arrows denotes columns with higher intensity while the blue arrows denotes columns with lower intensity.; Figure S3: HRTEM image of 40 nm LSC/(100)LSGM in the [001] zone axis. Thin dashed white line marks the interface. In the red rectangle is the image simulation of the interface. Simulation parameters was set to defocus of 27.00 nm and the sample thickness of 16.27 nm; Figure S4: Dark field image of LSC/(111)LSGM with selected $\vec{g}$ = 1 $\frac{1}{2}$$\frac{1}{2}$ near [0–11] beam direction. Red arrows highlight precipitate locations; Figure S5: Atomic resolution HAADF image of the LSC/(110)LSGM at the interface region (a) where the region taken for a intensity profile is marked by a red rectangle and the red arrow points the direction of the intensity line profile. Intensity line-profile is shown in (b) and here the interface position is indicated by a dashed red line; Figure S6: EELS spectra of the LSC/(100)LSGM (a), LSC/(110)LSGM (b) and LSC/(111)LSGM sample with the energy onset difference marked by red lines.; Table S1: Monoclinic cell (version 1): Lattice parameters a = 20.05000 nm, b = 4.01000 nm, c = 3.90000 nm, alpha = 90.0000 beta = 89.3000, gamma = 90.0000.; Table S2: Monoclinic cell (version 2): Lattice parameters a = 20.05000 nm, b = 4.01000 nm, c = 3.90000 nm, alpha = 90.0000 beta = 89.3000, gamma = 90.0000.; Table S3: Tetragonal cell (version 1): Lattice parameters a = 19.65750 nm, b = 3.93150 nm, c = 3.88910 nm, alpha = 90.0000 beta = 90.0000, gamma = 90.0000.; Table S4: Tetragonal cell (version 2): Lattice parameters a = 19.65750 nm, b = 3.93150 nm, c = 3.88910 nm, alpha = 90.0000 beta = 90.0000, gamma = 90.0000.
