# Supplementary material for: Structural Characterization of La0.6Sr0.4CoO3−δ Thin Films Grown on (100)-, (110)-, and (111)-Oriented La0.95Sr0.05Ga0.95Mg0.05O3−δ
Source: Materials (Basel). 2024 Apr 14;17(8):1802. doi: 10.3390/ma17081802 (PMC11050905; doi:10.3390/ma17081802)
Supplement: Supplementary file 1 [file materials-17-01802-s001.zip › materials-2918570-supplementary.pdf]

Article

Supporting information: Structural Characterization of $La_{0.6}Sr_{0.4}CoO_{3-\delta}$ Thin Films Grown on (100), (110) and (111) Oriented $La_{0.95}Sr_{0.05}Ga_{0.95}Mg_{0.05}O_{3-\delta}$

Sergej Ražnjević [1,*], Sandra Drev [2], Andreas E. Bumberger [3], Maxim N. Popov [4], Matthäus Siebenhofer [3,5,6], Christin Böhme [3], Zhuo Chen [1], Yong Huang [1], Christoph Riedl [3], Jürgen Fleig [3], Miran Čeh [2], Markus Kubicek [3], and Zaoli Zhang [1]

1 Erich Schmid Institute of Materials Science, Austrian Academy of Sciences, Jahnstraße 12, 8700 Leoben, Austria;
2 Center for Electron Microscopy and Microanalysis, Jožef Stefan Institute, Jamova cesta 39, 1000 Ljubljana, Slovenia;
3 Institute of Chemical Technologies and Analytics, TU Wien, Getreidemarkt 9, A-1060 Vienna, Austria;
4 Materials Center Leoben Forschung GmbH (MCL), Roseggerstraße 12, A-8700 Leoben, Austria;
5 Centre of Electrochemistry and Surface Technology, Viktor Kaplan-Straße 2, 2700 Wiener Neustadt;
6 Department of Nuclear Science and Engineering, Massachusetts Institute of Technology, 77 Massachusetts Avenue, 02141 Cambridge, U.S.
* Correspondence: sergejraznjevic@gmail.com

Citation: Ražnjević et al. Structural Characterization of $La_{0.6}Sr_{0.4}CoO_{3-\delta}$ Thin Films Grown on (100), (110) and (111) Oriented $La_{0.95}Sr_{0.05}Ga_{0.95}Mg_{0.05}O_{3-\delta}$. *Journal Not Specified* **2024**, *1*, 0. https://doi.org/

Received:
Revised:
Accepted:
Published:

Copyright: © 2024 by the authors. Submitted to *Journal Not Specified* for possible open access publication under the terms and conditions of the Creative Commons Attribution (CC BY) license (https://creativecommons.org/licenses/by/4.0/).

1. Supporting Information

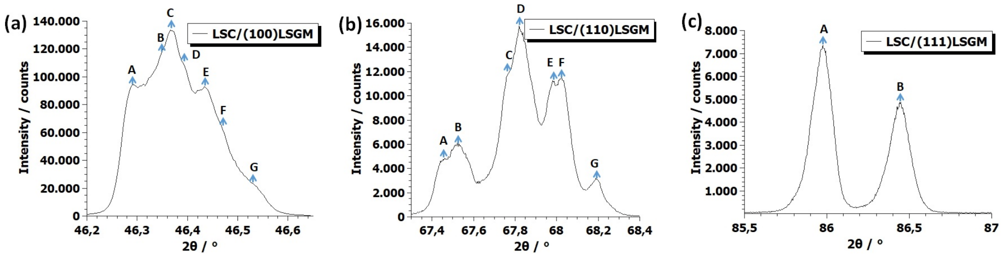

Figure S1. Close up view at the (a) (200), (b) (220) and (c) (222) reflections from the XRD θ-2θ scan.

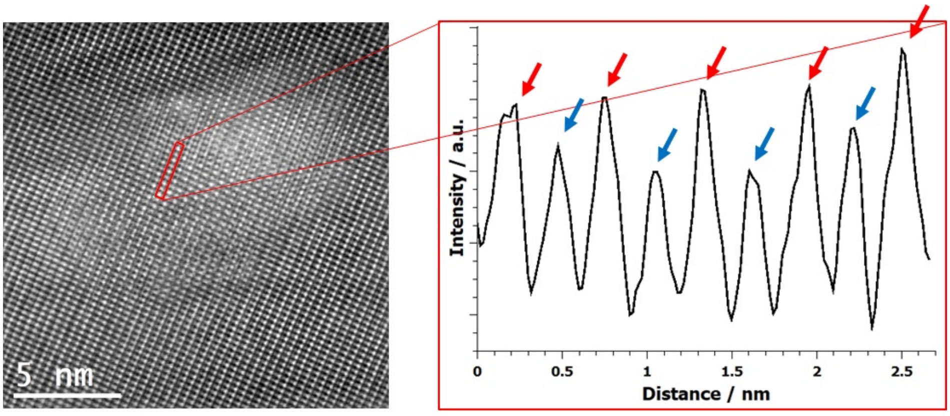

Figure S2. Average background subtraction filtered HAADF image (left) and the intensity line trace (right). In the line-trace red arrows denotes columns with higher intensity while the blue arrows denotes columns with lower intensity.

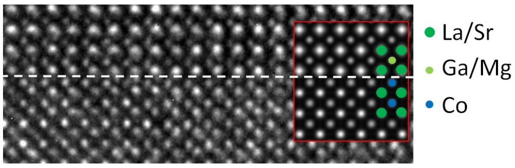

Figure S3. HRTEM image of 40nm LSC/(100)LSGM in the [001] zone axis. Thin dashed white line marks the interface. In the red rectangle is the image simulation of the interface. Simulation parameters was set to defocus of 27.00 nm and the sample thickness of 16.27 nm.

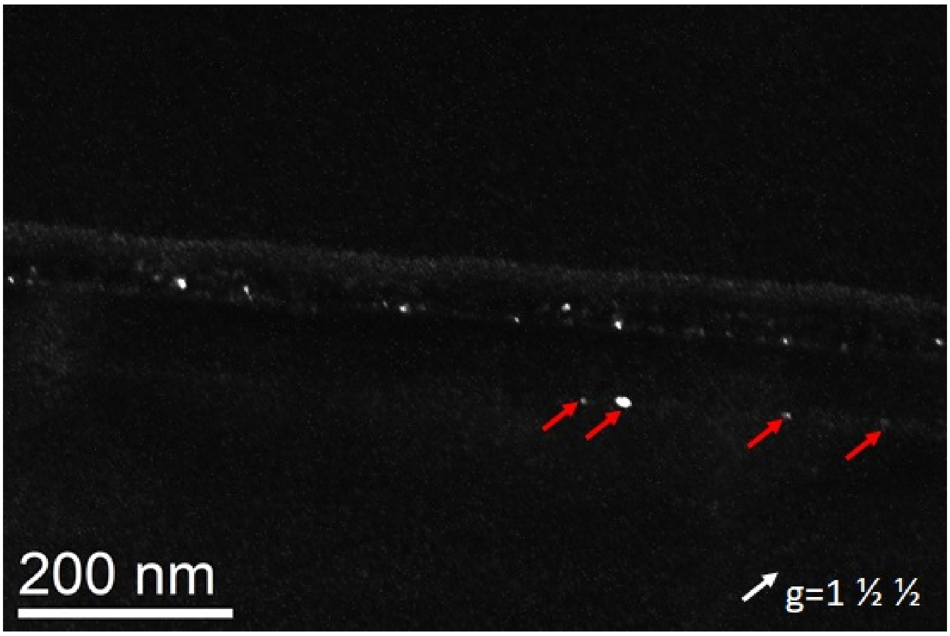

Figure S4. Dark field image of LSC/(111)LSGM with selected $\vec{g} = 1\ \frac{1}{2}\ \frac{1}{2}$ near [0-11] beam direction. Red arrows highlight precipitate locations.

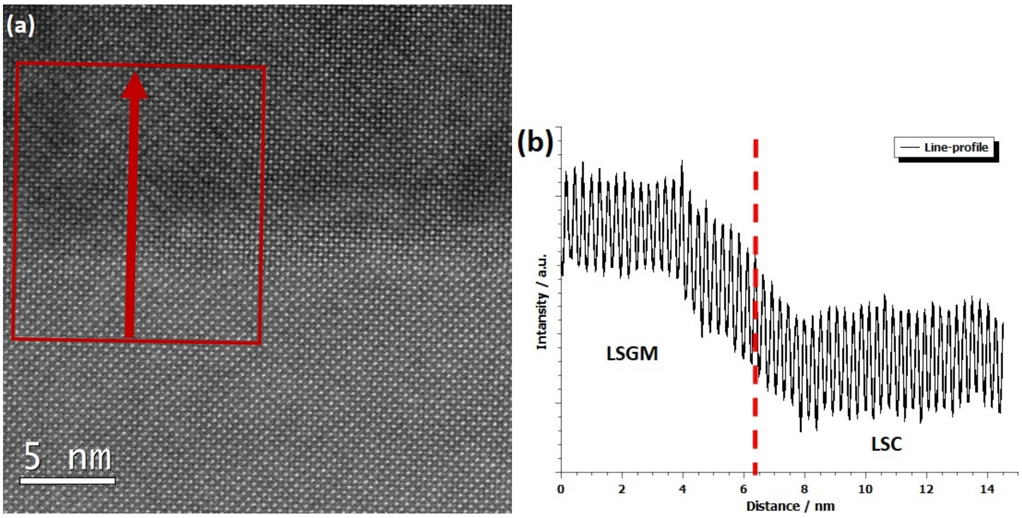

Figure S5. Atomic resolution HAADF image of the LSC/(110)LSGM at the interface region (a) where the region taken for a intensity profile is marked by a red rectangle and the red arrow points the direction of the intensity line profile. Intensity line-profile is shown in (b) and here the interface position is indicated by a dashed red line.

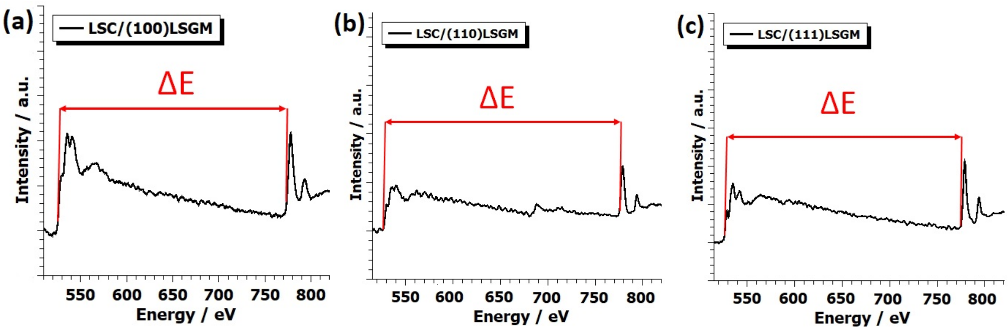

Figure S6. EELS spectra of the LSC/(100)LSGM (a), LSC/(110)LSGM (b) and LSC/(111)LSGM sample with the energy onset difference marked by red lines.

Table S**1.** Monoclinic cell (version 1): Lattice parameters a = 20.05000 nm, b = 4.01000 nm, c = 3.90000 nm, alpha = 90.0000 beta = 89.3000, gamma = 90.0000

Number	Atom	x	y	z	occ
1	La	0.40000	0.00000	0.00000	1
2	La	0.60000	0.00000	0.00000	1
3	La	0.80000	0.00000	0.00000	1
4	Sr	0.00000	0.00000	0.00000	1
5	Sr	0.20000	0.00000	0.00000	1
6	Co	0.10000	0.50000	0.50000	1
7	Co	0.30000	0.50000	0.50000	1
8	Co	0.50000	0.50000	0.50000	1
9	Co	0.70000	0.50000	0.50000	1
10	Co	0.90000	0.50000	0.50000	1
11	O	0.10000	0.50000	0.00000	1
12	O	0.10000	0.00000	0.50000	1
13	O	0.00000	0.50000	0.50000	1
14	O	0.30000	0.50000	0.00000	1
15	O	0.30000	0.00000	0.50000	1
16	O	0.20000	0.50000	0.50000	1
17	O	0.50000	0.50000	0.00000	1
18	O	0.50000	0.00000	0.00000	1
19	O	0.40000	0.50000	0.50000	1
20	O	0.70000	0.50000	0.00000	1
21	O	0.70000	0.00000	0.50000	1
22	O	0.60000	0.50000	0.50000	1
23	O	0.90000	0.50000	0.00000	1
24	O	0.90000	0.00000	0.50000	1
25	O	0.80000	0.50000	0.50000	1

Table S**2.** Monoclinic cell (version 2): Lattice parameters a = 20.05000 nm, b = 4.01000 nm, c = 3.90000 nm, alpha = 90.0000 beta = 89.3000, gamma = 90.0000

Number	Atom	x	y	z	occ
1	La	0.20000	0.00000	0.00000	1
2	La	0.60000	0.00000	0.00000	1
3	La	0.80000	0.00000	0.00000	1
4	Sr	0.00000	0.00000	0.00000	1
5	Sr	0.40000	0.00000	0.00000	1
6	Co	0.10000	0.50000	0.50000	1
7	Co	0.30000	0.50000	0.50000	1
8	Co	0.50000	0.50000	0.50000	1
9	Co	0.70000	0.50000	0.50000	1
10	Co	0.90000	0.50000	0.50000	1
11	O	0.10000	0.50000	0.00000	1
12	O	0.10000	0.00000	0.50000	1
13	O	0.00000	0.50000	0.50000	1
14	O	0.30000	0.50000	0.00000	1
15	O	0.30000	0.00000	0.50000	1
16	O	0.20000	0.50000	0.50000	1
17	O	0.50000	0.50000	0.00000	1
18	O	0.50000	0.00000	0.00000	1
19	O	0.40000	0.50000	0.50000	1
20	O	0.70000	0.50000	0.00000	1
21	O	0.70000	0.00000	0.50000	1
22	O	0.60000	0.50000	0.50000	1
23	O	0.90000	0.50000	0.00000	1
24	O	0.90000	0.00000	0.50000	1
25	O	0.80000	0.50000	0.50000	1

Table S**3.** Tetragonal cell (version 1): Lattice parameters a = 19.65750 nm, b = 3.93150 nm, c = 3.88910 nm, alpha = 90.0000 beta = 90.0000, gamma = 90.0000

Number	Atom	x	y	z	occ
1	La	0.40000	0.00000	0.00000	1
2	La	0.60000	0.00000	0.00000	1
3	La	0.80000	0.00000	0.00000	1
4	Sr	0.00000	0.00000	0.00000	1
5	Sr	0.20000	0.00000	0.00000	1
6	Co	0.10000	0.50000	0.50000	1
7	Co	0.30000	0.50000	0.50000	1
8	Co	0.50000	0.50000	0.50000	1
9	Co	0.70000	0.50000	0.50000	1
10	Co	0.90000	0.50000	0.50000	1
11	O	0.10000	0.50000	0.00000	1
12	O	0.10000	0.00000	0.50000	1
13	O	0.00000	0.50000	0.50000	1
14	O	0.30000	0.50000	0.00000	1
15	O	0.30000	0.00000	0.50000	1
16	O	0.20000	0.50000	0.50000	1
17	O	0.50000	0.50000	0.00000	1
18	O	0.50000	0.00000	0.00000	1
19	O	0.40000	0.50000	0.50000	1
20	O	0.70000	0.50000	0.00000	1
21	O	0.70000	0.00000	0.50000	1
22	O	0.60000	0.50000	0.50000	1
23	O	0.90000	0.50000	0.00000	1
24	O	0.90000	0.00000	0.50000	1
25	O	0.80000	0.50000	0.50000	1

Table S**4.** Tetragonal cell (version 2): Lattice parameters a = 19.65750 nm, b = 3.93150 nm, c = 3.88910 nm, alpha = 90.0000 beta = 90.0000, gamma = 90.0000

Number	Atom	x	y	z	occ
1	La	0.20000	0.00000	0.00000	1
2	La	0.60000	0.00000	0.00000	1
3	La	0.80000	0.00000	0.00000	1
4	Sr	0.00000	0.00000	0.00000	1
5	Sr	0.40000	0.00000	0.00000	1
6	Co	0.10000	0.50000	0.50000	1
7	Co	0.30000	0.50000	0.50000	1
8	Co	0.50000	0.50000	0.50000	1
9	Co	0.70000	0.50000	0.50000	1
10	Co	0.90000	0.50000	0.50000	1
11	O	0.10000	0.50000	0.00000	1
12	O	0.10000	0.00000	0.50000	1
13	O	0.00000	0.50000	0.50000	1
14	O	0.30000	0.50000	0.00000	1
15	O	0.30000	0.00000	0.50000	1
16	O	0.20000	0.50000	0.50000	1
17	O	0.50000	0.50000	0.00000	1
18	O	0.50000	0.00000	0.00000	1
19	O	0.40000	0.50000	0.50000	1
20	O	0.70000	0.50000	0.00000	1
21	O	0.70000	0.00000	0.50000	1
22	O	0.60000	0.50000	0.50000	1
23	O	0.90000	0.50000	0.00000	1
24	O	0.90000	0.00000	0.50000	1
25	O	0.80000	0.50000	0.50000	1
